# Simvastatin Inhibits NLRP3 Inflammasome Activation and Ameliorates Lung Injury in Hyperoxia-Induced Bronchopulmonary Dysplasia via the KLF2-Mediated Mechanism

**DOI:** 10.1155/2022/8336070

**Published:** 2022-04-25

**Authors:** Xinye Wang, Ran Huo, Zhongjie Liang, Congcong Xu, Tingting Chen, Jingjing Lin, Luyao Li, Wei Lin, Bingting Pan, Xiaoqin Fu, Shangqin Chen

**Affiliations:** ^1^Department of Neonatology, The Second Affiliated Hospital, Yuying Children's Hospital of Wenzhou Medical University, Zhejiang, China; ^2^Department of Pediatric, Zhejiang Provincial People's Hospital, People's Hospital of Hangzhou Medical College, Hangzhou, China

## Abstract

Bronchopulmonary dysplasia (BPD) is a chronic lung disease commonly found in premature infants. Excessive inflammation and oxidative stress contribute to BPD occurrence and development. Simvastatin, as an inhibitor of HMG-CoA reductase, has been reported to have antioxidative and anti-inflammatory effects. However, its effect and possible mechanisms in hyperoxia-induced lung injury are rarely reported. In this study, *in vivo* and *in vitro* experiments were conducted to investigate whether simvastatin could ameliorate hyperoxia-induced lung injury and explore its potential mechanism. For the *in vivo* study, simvastatin could improve alveolar development after hyperoxic lung injury and reduce hyperoxic stress and inflammation. The *in vitro* study revealed that simvastatin can reduce inflammation in A549 cells after high-oxygen exposure. Simvastatin suppressed NLRP3 inflammasome activation and played anti-inflammatory and antioxidant roles by increasing KLF2 (Krüppel-like factor 2) expression. *In vitro* experiments also revealed that these effects of simvastatin were partially reversed by KLF2 shRNA, indicating that KLF2 was involved in simvastatin effects. In summary, our findings indicate that simvastatin could downregulate NLRP3 inflammasome activation and attenuate lung injury in hyperoxia-induced bronchopulmonary dysplasia via KLF2-mediated mechanism.

## 1. Introduction

Bronchopulmonary dysplasia (BPD) is a chronic lung disease of preterm infants [1]. According to prior research, genetic susceptibility, premature delivery, mechanical ventilation, infection, oxidative stress injury, and lung inflammation may jointly participate in BPD occurrence and development [2]. In recent years, as neonatal intensive care has improved, the survival rates of very low-birth weight infants (<1500 g)/extremely low-birth weight infants (<1000 g) have increased but BPD incidence has also significantly increased [3]. Although BPD has been clinically prevented and treated by controlling the oxygen concentration, caffeine, and vitamin A, the therapeutic effect and prognosis of BPD are still not satisfactory. As a result, developing other innovative and effective preventive or therapeutic measures to reduce mortality rates is a great challenge for perinatal medicine.

Since premature infants are sensitive to excessive oxidative stress, continuous exposure to a high-oxygen environment produces excessive reactive oxygen species, activating specific inflammatory cells and ultimately causing lung damage and cell death [4, 5]. Studies have indicated that excessive inflammatory factors, such as IL-6, TNF-*α*, and IL-1*β*, have been implicated in hyperoxic lung injury pathogenesis [6–9]. Inflammasome is a major component of innate immunity, and leucine-rich repeat protein 3 (NLRP3) inflammasome is the most studied [10, 11]. NLRP3 inflammasome mainly comprises NLRP3, caspase adaptor (ASC), and caspase-1 [12]. Recent studies have demonstrated that NLRP3 is involved in many common lung diseases, such as acute lung injury and pulmonary fibrosis [13–15]. In addition, Chen et al. found that NLRP3 can be activated in the BPD model exposed to 85% oxygen and plays a critical role in inflammation and alveolarization [16]. Therefore, NLRP3 inflammasome is involved in BPD pathogenesis and is expected to become a new target for treating BPD in the future.

Krüppel-like factor 2 (KLF2), also referred to as lung Krüppel-like factor (LKLF), is a member of the zinc finger Krüppel-like transcription factor family and is involved in cell differentiation and tissue development [17]. KLF2 is mainly expressed in lung tissue and is required for normal lung development [18, 19]. In addition, KLF2 is a known inflammation regulator [20]. KLF2 could negatively regulate the expression of inflammatory cytokines and the production of adhesion molecules [21]. It has recently been discovered to participate in the occurrence and development of various lung diseases, including acute lung injury, asthma, and chronic obstructive pulmonary disease [18, 22–25]. Lung inflammation induced by lipopolysaccharide (LPS) or influenza A H1N1 virus could lead to reduced KLF2 [22]. However, the expression and effects of KLF2 on hyperoxic lung injury have not yet been investigated.

As an HMG-CoA reductase inhibitor, simvastatin is a widely used drug for treating dyslipidemia and cardiovascular diseases [26–29]. In addition, simvastatin demonstrates vascular protective effects by inducing KLF2 expression [30]. Recent clinical evidence suggests that simvastatin exhibits additional pharmacological effects, such as antioxidant and anti-inflammatory activities, as well as endothelial protection [31]. Liu et al. discovered that simvastatin exerts its therapeutic effect in rats with hepatic ischemia-reperfusion injury via a KLF2-dependent mechanism [32]. In addition, Sun et al. found that simvastatin improved human endothelial cell barrier function and reduced lipopolysaccharide-induced lung and systemic inflammation based on *in vivo* and *in vitro* experiments [33]. Consequently, we hypothesized that simvastatin could protect against hyperoxia-induced bronchopulmonary dysplasia by suppressing NLRP3 activation and acting as an anti-inflammatory and antioxidant agent by upregulating KLF2 expression.

## 2. Materials and Methods

### 2.1. Hyperoxia-Induced Lung Injury

All Sprague-Dawley rats used in animal experiments were obtained from the animal center of the Chinese Academy of Sciences (Shanghai, China), following the guidelines for the use of experimental animal care issued by the National Institutes of Health and approved by the experimental ethics committee of Wenzhou Medical University. Adult rats were housed in the laboratory animal center with humidity and temperature controlled at 60 ± 10% and 23 ± 2°C, respectively. The pups (no distinction between males and females) were randomly divided into four experimental groups and were returned to nursing cages within 6 h after birth: normoxia (NO) group, normoxia + simvastatin (NS) group, hyperoxia (HO) group, and hyperoxia + simvastatin (HS) group. Based on the dosages of simvastatin used in other models, the intermediate dose of 5 mg/kg was selected in our study to examine its effects on the hyperoxia lung injury in neonatal SD rats [34–36]. Simvastatin dissolved in corn oil (5 mg/kg, MCE, USA) was intraperitoneally injected into the pups of NS and HS groups on P0–P7. The pups in NO and HO groups received the same volume of vehicle corn oil (Aladdin, Shanghai, China).

The pups in the normoxia group received 21% oxygen for seven days, whereas those in the hyperoxia group received 80–85% oxygen. The plexiglass chamber flow-through system was employed to maintain a constant 80–85% oxygen, and the O_2_ level was monitored using an O_2_ analyzer. For seven days, we rotated the nursing dams between hyperoxia and normoxia groups every 24 h to protect nursing mothers from O_2_ toxicity.

### 2.2. Lung Histological and Morphometric Analyses

The pups were sacrificed using 1% pentobarbital by intraperitoneal injection. After ligating the right bronchus, the left lungs were perfused with PBS and inflated to 20 cmH_2_O pressure with 4% paraformaldehyde (PFA) and then preserved and fixed in 4% PFA for 48 h. The paraffin-embedded lung tissues were cut into 5 *μ*m sections, stained with hematoxylin and eosin (HE) (Beyotime, China), and morphologically analyzed under a microscope (Nikon, Japan). The radial alveolar count (RAC), mean linear intercept (MLI), and mean alveolar diameter (MAD), which were utilized to determine the alveolarization degree, were blindly assessed by investigators. There were at least 6 rats per group and at least 5 nonoverlapping HE pictures per rat. RAC was obtained by calculating the number of alveoli passing through the vertical line from the edge of terminal bronchioles to the nearest pleura or mediastinum. MAD was defined as the average alveolar diameter. Five lines were randomly drawn in each region, and the number of alveoli passing across each line was MLI.

### 2.3. Water Content in the Lung

The moisture content of the lungs is determined using lung tissues from the right lower lobe. To obtain the wet weight, the lobes were isolated and wrapped in a preweighted dry and clean tube. The lung tissue was placed at 80°C electric oven and dried for 24 h to obtain a dry weight. To determine lung tissue edema, the *W*/*D* ratio was calculated using the formula: (wet weight − dry weight)/humidity∗100%.

### 2.4. Cell Culture

A549 cell lines were maintained in DMEM/F-12 containing 10% fetal bovine serum (FBS) (Gibco) and cultured in an incubator with 5% CO_2_ at 37°C. The experiments were performed until the cells filled approximately 80% of the bottle bottom. The normoxia (NO) group continued to be cultured in an incubator with 5% CO_2_, whereas the hyperoxia group was cultured in a special incubator containing 85% O_2_ and 5% CO_2_.

### 2.5. Cell Transfection and Grouping

When cells reached an ~80% confluence, various plasmids (Sangon Biotech, Shanghai, China) were transfected into A549 cells according to instructions of using the Lipofectamine™ 2000 Reagent (Invitrogen, California, USA). After adding the mixture of transfection reagent, plasmid, and Opti culture medium, the cells were cultured in a 37°C incubator for 30 min before replacing the old medium with a normal medium. For siRNA transfection, the corresponding scrambled sequence was employed as a control in the normal group to eliminate the impact of transfection on the cells. The complementary RNAi oligos were annealed and ligated into pSilencer-GFP, which is a bicistronic plasmid that expresses shRNA and eGFP, as previously described by Konishi et al. [37]. In addition, the transfection efficiency was 40%~45%. Accordingly, cells were grouped into the normoxia group (NO), normoxia + KLF2 shRNA group (NO + KLF2 shRNA), hyperoxia group (HO), hyperoxia + KLF2 shRNA group (HO + KLF2shRNA), normoxia + simvastatin group (NS), hyperoxia + simvastatin group (HS), and hyperoxia + simvastatin + KLF2 shRNA group (HS + KLF2shRNA).

### 2.6. Cell Counting Assay

To determine the appropriate high-oxygen treatment time and doses of simvastatin, we exposed the cells to 85% O_2_ for 4, 8, 12, 24, 36, and 48 h and added different simvastatin doses (2.5, 5, 10, 15, and 20 *μ*M) before high-oxygen treatment. The cell viability was measured using Cell Counting Kit-8 assays (CCK8) (C0038, Beyotime, China). After 48 h incubation, 10 *μ*L CCK-8 solution was added to each well and incubated at 37°C for 1 h in the dark. The OD value was measured at 450 nm, and cell viability of each group was calculated with reference to the normoxia group.

### 2.7. Western Blotting

First, the tissues were homogenized using protein lysates consisting of RIPA lysis buffer (P0013B; Beyotime) and a protease and phosphatase inhibitor cocktail (P1048; Beyotime) and centrifuged at 12000 rpm for 30 min at 4°C. We then quantified the proteins using the BCA kit (P0010S; Beyotime). Equal mass proteins (50ug) were separated by 10% or 12.5% Tris-glycine gels in SDS-PAGE. After being transferred to PVDF membranes (Merck KGaA, Darmstadt, Germany) at 300 mA for 2 h, the membranes were blocked with 5% skim milk for 3 h at room temperature and incubated with appropriate primary antibodies, including KLF2 (1 : 1000, A16480, ABclonal, Wuhan, China), NLRP3 (1 : 1000, A12694, ABclonal, Wuhan, China), caspase-1/P20/P10 (1 : 1000, 22915-1AP; ProteinTech), IL-1*β* (1 : 1000, A1112, ABclonal, Wuhan, China), ASC (1 : 1000, 340097, ZENBIO, Chengdu, China), and *β*-actin (1 : 5000, AF7018, Affinity Biosciences, Cincinnati, OH, USA) overnight at 4°C. On the second day, the membranes were washed three times in Tris-buffered saline and Tween 20, followed by their incubation with appropriate secondary antibodies for 2 h at room temperature: goat anti-rabbit IgG (1 : 5000, SE134, Solarbio, Beijing, China) or goat anti-mouse IgG (1 : 5000, SE131, Solarbio, Beijing, China). The protein bands were detected using enhanced chemiluminescence reagents (Epizyme Biotech, shanghai, China) through the ChemiDoc XRS + Imaging System (Bio-Rad, Hercules, CA, USA). All protein bands were calculated using Image Lab 5.0 software (Bio-Rad, Hercules, CA, USA).

### 2.8. Immunofluorescence

After drying overnight at 37°C, the 5 *μ*m lung tissue sections were deparaffinized using a gradient series of xylene and ethanol. Antigen retrieval was performed by microwave heating the sections for 20 min in 10 mM citric acid buffer (pH 6.0). After three washes with 1x PBS, the sections were blocked using 10% goat serum albumin for 1 h. KLF2 (1 : 100, A16480, ABclonal, Wuhan, China) and Ki67 (1 : 100, A2094, ABclonal, Wuhan, China) were diluted in 10% goat serum albumin, and 30 *μ*L was added to the sections (overnight at 4°C). The following day, the sections were incubated at room temperature for 2 h with Alexa Fluor-488 goat anti-rabbit IgG (1 : 200; AB150077; Abcam) and Alexa Fluor-555 goat anti-rabbit IgG (1 : 200; AB150078; Abcam). Finally, the sections were treated with a mounting medium containing 4′,6-diamidino-2-phenylindole (Solarbio, Beijing, China) and the images were obtained using a scanning microscope (C1; Nikon, Tokyo, Japan).

### 2.9. Enzyme-Linked Immunosorbent Assays (ELISA)

After ligating the right bronchus, 200 *μ*L of PBS was injected through the tracheal tube to lavage the left lung three times. The lavage fluid was recovered and centrifuged at 3000 rpm for 10 minutes to obtain bronchoalveolar lavage fluid (BALF). Then, the TNF-*α*, IL-6, and IL-1*β* levels in BALF were determined by the rat cytokine enzyme-linked immunosorbent assay (ELISA) kit, following manufacturer's instructions (Multi Sciences Lianke Biotech, Hangzhou, China).

### 2.10. Assessment of Oxidative Stress in Lung Tissues

Superoxide dismutase (SOD) and glutathione (GSH) levels in lung tissue were measured using a commercially available kit (BC0170/BC1175, Solarbio, Beijing, China). A certain amount of lung tissue was obtained, the extract was added proportionally, and ice bath homogenization was conducted. The specific operation and result analysis were conducted following kit instructions.

### 2.11. Statistical Analysis

The data from experiments that were performed at least three independent times are presented as mean ± SD. GraphPad Prism 8.0 (GraphPad Software, San Diego, USA) and SPSS Statistics version 19.0 (SPSS Inc., Chicago, IL) were used for statistical analysis. Differences between groups were analyzed using one- and two-way ANOVA followed by Bonferroni post hoc test. *P* < 0.05 values were considered statistically significant.

## 3. Results

### 3.1. Effect of Simvastatin on Pulmonary Alveolar Simplification in the Lung

**Figure 1:**
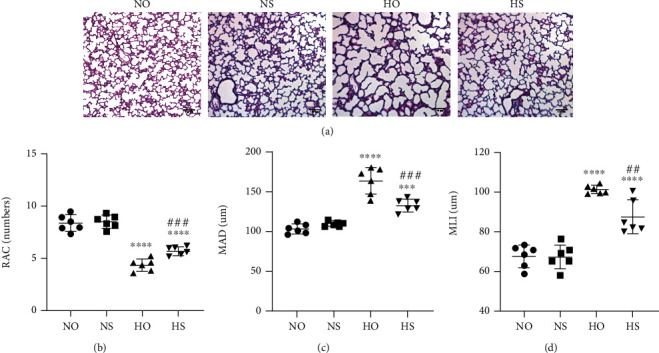
HE staining in the images and assessment of RAC, MAD, and MLI revealed that hyperoxia exposure led to marked alveolar simplification. In addition, simvastatin treatment could attenuate lung morphological changes. (a) HE staining (light microscopy, ×100) of lung tissue slides from each group. Scale bar = 100 *μ*m. (b–d) Semiquantitative pathology determination of RAC, MAD, and MLI in lung tissues. The values are mean ± SD; *n* = 6, analyzed by two-way ANOVA with the Bonferroni post hoc test. ^∗∗∗^*P* < 0.001 and ^∗∗∗∗^*P* < 0.0001 versus the normoxia group; ^##^*P* < 0.01 and ^###^*P* < 0.001 versus the hyperoxia group.

Figure 1 illustrates the lung morphology using HE staining. In normoxia and normoxia + simvastatin groups, the lung exhibited complete lung structures with normal alveolar epithelium and alveolar septum (Figure 1(a)). However, after seven days of hyperoxia exposure, the lungs of hyperoxia-exposed rats were significantly simplified, MLI and MAD of per unit area increased, and RAC decreased (Figures 1(b)–1(d)). These data manifested that prolonged exposure to hyperoxia can increase alveolar damage, cause alveolar simplification, and delay lung development. Compared with that in the hyperoxia group, the alveolar simplification in the hyperoxia + simvastatin group was significantly improved. Compared with that of the hyperoxia group, RAC of the hyperoxia + simvastatin group increased, while MLI and MAD decreased. These findings indicated that simvastatin treatment could combat hyperoxia-induced lung injury and partially restore hyperoxia-induced alveolar simplification.

### 3.2. Simvastatin Maintains KLF2 Expression

**Figure 2:**
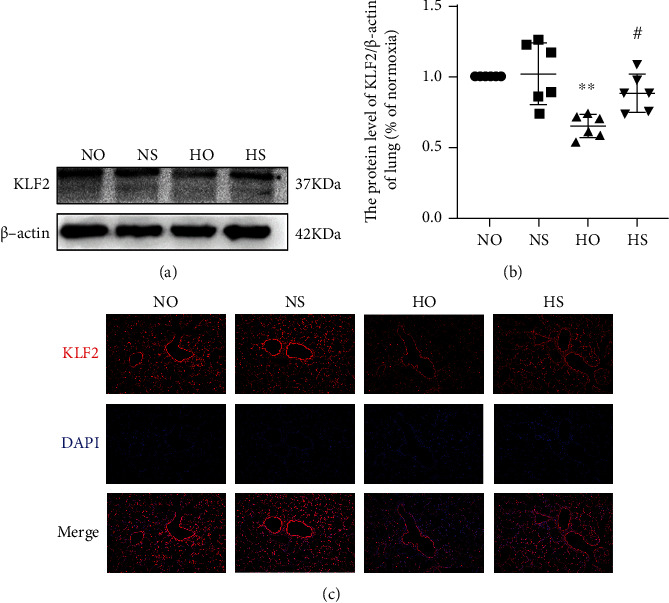
KLF2 levels in lung were decreased after hyperoxia. Simvastatin treatment significantly reversed KLF2 reduction after hyperoxia-induced lung injury. (a) KLF2 levels in lung tissues as determined by Western blotting. (b) Analyses of KLF2 levels based on Western blot results (normalized to *β*-actin levels). The values are the mean ± SD; *n* = 6, analyzed by two-way ANOVA with the Bonferroni post hoc test, ^∗∗^*P* < 0.01 versus the normoxia group; ^#^*P* < 0.05 versus the hyperoxia group. (c) IF staining image of KLF2 in lung tissue (light microscopy, ×100). Scale bar represents 200 *μ*m.

The KLF2 level in the lung was determined by Western blotting (Figures 2(a) and 2(b)). KLF2 expression was decreased in the hyperoxia group compared with the normoxia and normoxia + simvastatin groups. However, the hyperoxia + simvastatin group had a higher KLF2 level than hyperoxia group and there was no statistically significant difference between normoxia and normoxia + simvastatin groups in KLF2 levels. These results indicated that hyperoxic injury reduces KLF2 expression in lungs while simvastatin could reverse hyperoxic effects on KLF2. Then, we used immunofluorescence to further explore the changes of KLF2 expression. We found that immunofluorescence results are consistent with those of Western blotting (Figure 2(c)), hyperoxia could reduce KLF2 expression, and simvastatin could reverse this effect.

### 3.3. Simvastatin May Decrease Oxidative Stress and Inflammation Infiltration in Neonatal Rats

**Figure 3:**
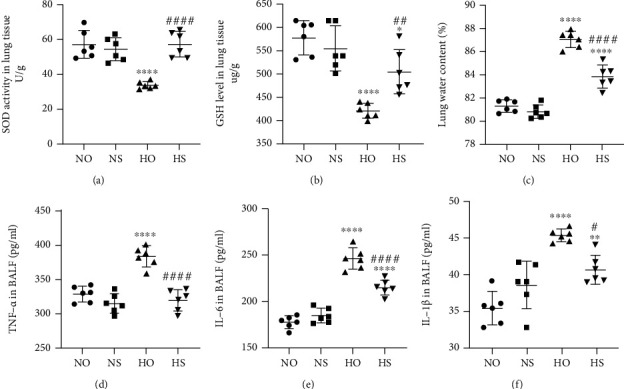
Effect of simvastatin on oxidative stress and inflammation in lung tissues. (a) SOD activity in various lung tissues. The values are demonstrated as the mean ± SD; *n* = 6, analyzed by two-way ANOVA with the Bonferroni post hoc test, ^∗∗∗∗^*P* < 0.0001 versus the normoxia group; ^####^*P* < 0.0001 versus the hyperoxia group. (b) GSH activity in different lung tissues. The values are displayed as the mean ± SD; *n* = 6, analyzed by two-way ANOVA with the Bonferroni post hoc test, ^∗^*P* < 0.05 and ^∗∗∗∗^*P* < 0.0001 versus the normoxia group; ^##^*P* < 0.01 versus the hyperoxia group. (c) The ratio of wet and dry weight is calculated in each group. The values are illustrated as the mean ± SD; *n* = 6, ^∗∗∗∗^*P* < 0.0001 versus the normoxia group; ^####^*P* < 0.0001 versus the hyperoxia group. (d–f) TNF-*α*, IL-6, and IL-1*β* levels in BALF. The values are manifested as the mean ± SD; *n* = 6, analyzed by two-way ANOVA with the Bonferroni post hoc test, ^∗∗^*P* < 0.01 and ^∗∗∗∗^*P* < 0.0001 versus the normoxia group; ^#^*P* < 0.05 and ^####^*P* < 0.0001 versus the hyperoxia group.

Oxidative stress expression was determined using available kits. As illustrated in Figures 3(a) and 3(b), the hyperoxia group had lower SOD and GSH activities than the normoxia group. However, SOD and GSH activities increased under simvastatin treatment in hyperoxia + simvastatin. To evaluate the effects of simvastatin on hyperoxia-induced inflammation in neonatal rats, we measured the lung water content and used ELISA kits to determine the expression of several inflammatory markers (TNF-*α*, IL-1*β*, and IL-6). As demonstrated in Figure 3(c), the ratio of wet and dry weight is calculated in each group; the water content was increased in the hyperoxia group but decreased following simvastatin treatment. As presented in Figures 3(d) and 3(e), TNF-*α* levels in the lung of the hyperoxia group were higher than those of the normoxia group. Conversely, the simvastatin treatment reversed the increase of TNF-*α* on hyperoxic inducement. Similar changes in IL-6 and IL-1*β* levels were found. Our findings indicated that simvastatin treatment significantly reduced lung edema in acute stages of hyperoxia lung injury, implying that simvastatin ameliorated oxidative stress and hyperoxia-induced inflammation infiltration in the lungs of neonatal rats.

### 3.4. Effect of Simvastatin on the NLRP3 Inflammasome Signaling Pathway in Hyperoxia-Induced Bronchopulmonary Dysplasia

**Figure 4:**
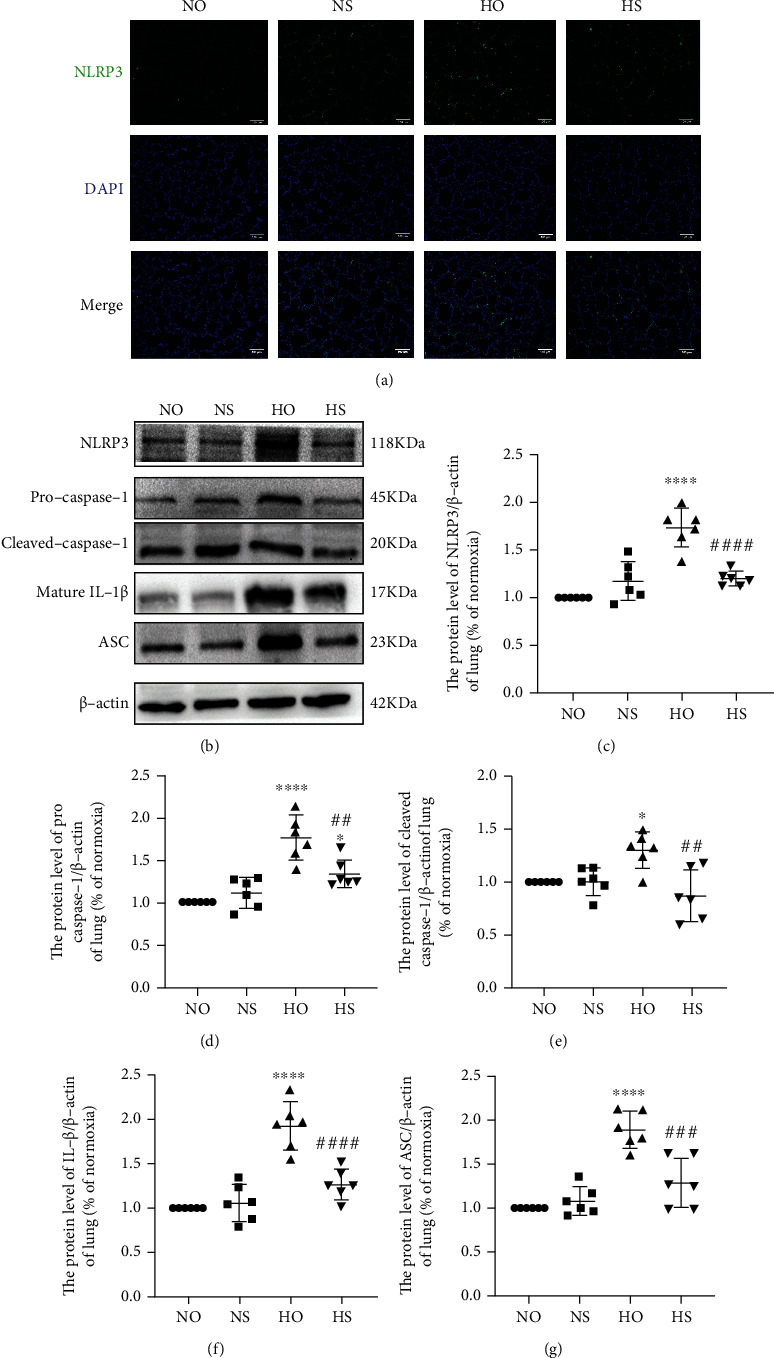
Hyperoxia exposure can activate NLRP3 inflammasome. Simvastatin treatment ameliorated this hyperoxia-induced change. (a) IF staining images of NLRP3 in the lungs of each group (microscopy, ×200). Scale bars = 100 *μ*m. (b) Western blotting results of NLRP3, pro-caspase-1, cleaved caspase-1, mature IL-1*β*, and ASC in different lung tissues. (c–g) The quantification of protein levels of NLRP3, pro-caspase-1, cleaved caspase-1, mature IL-1*β*, and ASC (normalized with *β*-actin). The values are the mean ± SD; *n* = 6, analyzed by two-way ANOVA with the Bonferroni post hoc test, ^∗^*P* < 0.05 and ^∗∗∗∗^*P* < 0.0001 versus the normoxia group; ^##^*P* < 0.01, ^###^*P* < 0.001, and ^####^*P* < 0.0001 versus the hyperoxia group.

By Western blotting and immunofluorescence, we quantified the expression of NLRP3, pro-caspase-1, cleaved caspase-1, mature IL-1*β*, and ASC in the lungs to evaluate the influence of simvastatin on NLRP3 inflammasome. Compared with those in the normoxia group, NLRP3 levels increased in the hyperoxia group, which could be partly reversed by simvastatin treatment (Figure 4). Similar changes were observed in the protein levels of pro-caspase-1, cleaved caspase-1, mature IL-1*β*, and ASC.

### 3.5. KLF2 Plays a Positive Role in Hyperoxia-Induced Lung Injury

**Figure 5:**
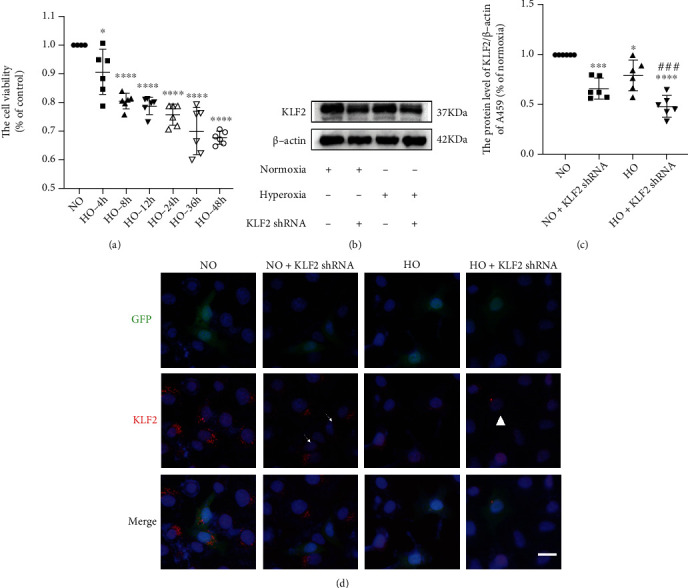
Hyperoxia and silencing of KLF2 significantly reduced KLF2 expression. (a) A549 cells were treated from 4 to 48 h, and cell viability was assessed using CCK8, *n* = 6, analyzed by one-way ANOVA followed by Tukey's post hoc test, ^∗^*P* < 0.05 and ^∗∗∗∗^*P* < 0.0001 versus the normoxia group. (b) Western blotting results of KLF2 in A549 cells. (c) Analyses of KLF2 expression (normalized to *β*-actin), *n* = 6, analyzed by two-way ANOVA with the Bonferroni post hoc test, ^∗^*P* < 0.05, ^∗∗∗^*P* < 0.001, and ^∗∗∗∗^*P* < 0.0001 versus the normoxia group; ^###^*P* < 0.001 versus the hyperoxia group. (d) IF staining images of KLF2 in each group (microscopy, ×400). Scale bars = 25 *μ*m.

**Figure 6:**
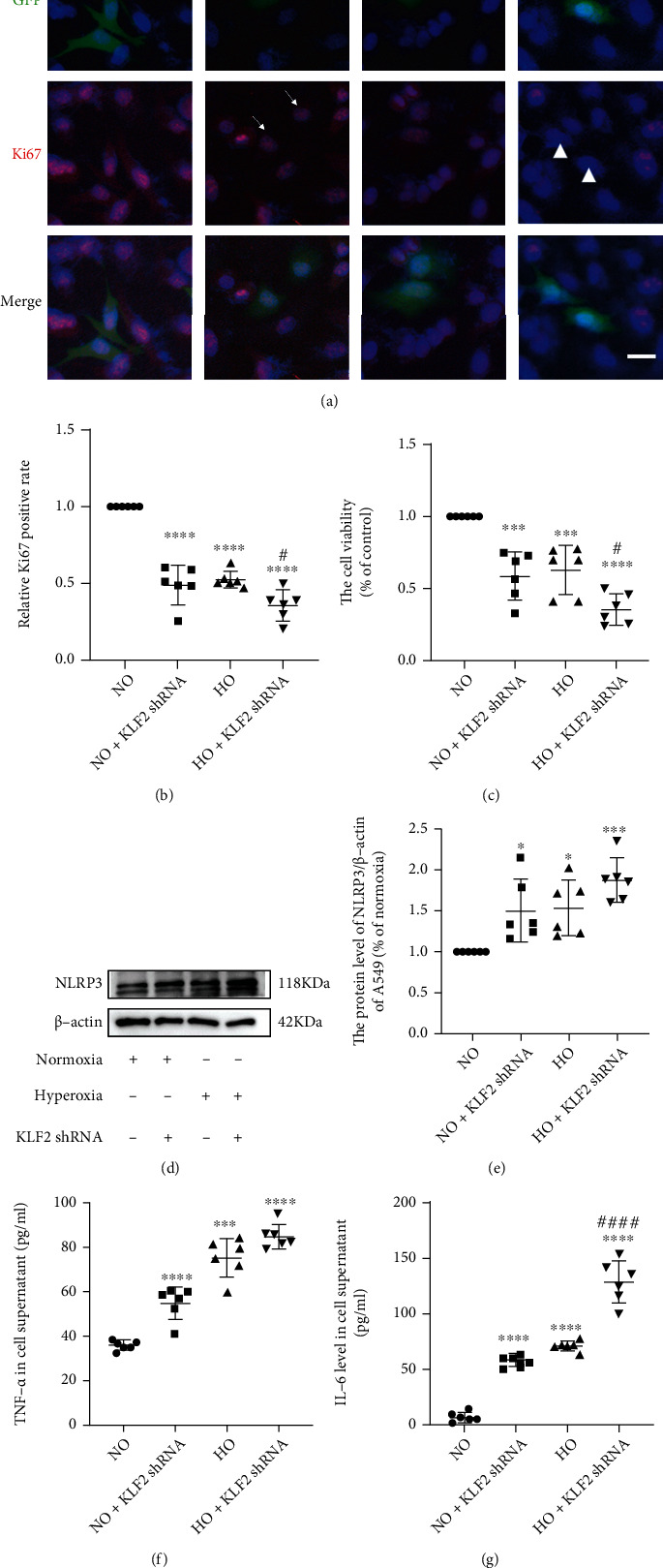
KLF2 shRNA reduced the cell viability of A549 and increased the expression of NLRP3 protein and inflammatory cytokines. (a) IF staining images of Ki67 in each group (microscopy, ×400). Scale bars = 25 *μ*m. (b) Quantitative analysis of Ki67^+^ cells of A549 cells, *n* = 6, analyzed by two-way ANOVA with the Bonferroni post hoc test, ^∗∗∗∗^*P* < 0.0001 versus the normoxia group, ^#^*P* < 0.05 versus the hyperoxia group. (c) Cell viability was determined using CCK8, *n* = 6, analyzed by two-way ANOVA with the Bonferroni post hoc test, ^∗∗∗^*P* < 0.001 and ^∗∗∗∗^*P* < 0.0001 versus the normoxia group, ^#^*P* < 0.05 versus the hyperoxia group. (d) Western blotting results of NLRP3 in A549 cells. (e) Analyses of NLRP3 expression (normalized to *β*-actin). *n* = 6, analyzed by two-way ANOVA with the Bonferroni post hoc test, ^∗^*P* < 0.05 and ^∗∗∗^*P* < 0.001 versus the normoxia group. (f–g) TNF-*α* and IL-6 levels in cells of different groups. *n* = 6, analyzed by two-way ANOVA with the Bonferroni post hoc test, ^∗∗∗^*P* < 0.001 and ^∗∗∗∗^*P* < 0.0001 versus the normoxia group. ^####^*P* < 0.0001 versus the hyperoxia group.

To further verify and explore the effect of simvastatin and its specific mechanism, we conducted *in vitro* experiments using A549 cell lines. Firstly, CCK8 analysis was used to select the optimal timing for hyperoxia. As indicated in Figure 5(a), 4 h after hyperoxia exposure, the viability of A549 cells begins to reduce and cell survival after 48 h of exposure remains more than 50%. As a result, 48 h of exposure was employed in all subsequent cell experiments. Based on the above *in vivo* studies, we hypothesized that simvastatin protects against hyperoxic lung injury via the KLF2 mechanism. To further explore this possibility, we used KLF2 shRNA for the *in vitro* study. To investigate the role of KLF2 in hyperoxic lung injury, we established two groups: normoxia + KLF2 shRNA group (NO + KLF2 shRNA) and hyperoxia + KLF2 shRNA group (HO + KLF2shRNA). As displayed in Figures 5(b)–5(d), KLF2 shRNA and hyperoxia could decrease KLF2 expression. CCK8 analyses and cellular immunofluorescence staining of Ki67 were used to evaluate the proliferation ability of cells. As demonstrated in Figures 6(a)–6(c), KLF2 plays a positive role in hyperoxia-induced lung injury. Silencing KLF2 with KLF2-specific shRNA aggravates the damage to hyperoxia-caused cell viability. In addition, as illustrated in Figures 6(d)–6(g), KLF2 shRNA and hyperoxia could increase the expression of NLRP3 protein and inflammatory cytokines in A549 cells.

### 3.6. Simvastatin Elicits Anti-Inflammatory Effects on Hyperoxia-Induced A549 Cell Injury via the KLF2-Mediated Mechanism

**Figure 7:**
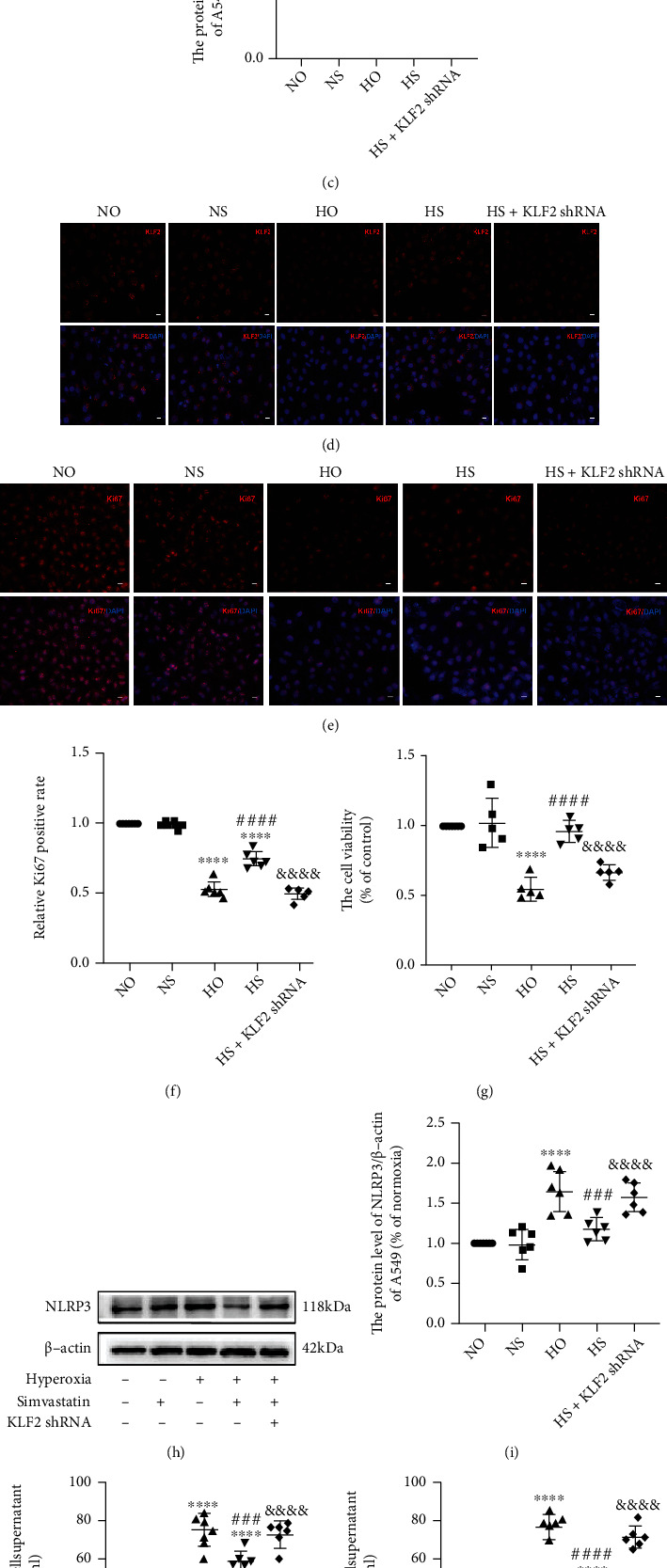
KLF2 shRNA could significantly block the protective effect of simvastatin. (a) The cell viability of different simvastatin concentrations, *n* = 6, analyzed by two-way ANOVA with the Bonferroni post hoc test, ^∗∗^*P* < 0.01 and ^∗∗∗^*P* < 0.001 versus the normoxia group, ^###^*P* < 0.001 versus the hyperoxia group. (b) The protein level of KLF2 was evaluated by Western blotting in A549 cells. (c) Analyses of KLF2 expression (normalized to *β*-actin), *n* = 6, analyzed by two-way ANOVA with the Bonferroni post hoc test, ^∗∗∗^*P* < 0.001 versus the normoxia group, ^####^*P* < 0.0001 versus the hyperoxia group, ^&&&&^*P* < 0.0001 versus the hyperoxia + simvastatin (HS) group. (d, e) IF staining images of Ki67 and KLF2 in each group (microscopy, ×400). Scale bars = 25 *μ*m. (f) Quantitative analysis of Ki67^+^ cells of A549 cells, n =6, analyzed by two-way ANOVA with the Bonferroni post hoc test, ^∗∗∗∗^*P* < 0.0001 versus the normoxia group, ^####^*P* < 0.0001 versus the hyperoxia group, ^&&&&^*P* < 0.0001 versus the hyperoxia + simvastatin group (HS). (g) The cell viability of different groups, *n* = 5, analyzed by two-way ANOVA with the Bonferroni post hoc test, ^∗∗∗∗^*P* < 0.0001 versus the normoxia group, ^####^*P* < 0.0001 versus the hyperoxia group, ^&&&&^*P* < 0.0001 versus the hyperoxia + simvastatin group (HS). (h) The protein level of NLRP3 was evaluated using Western blotting in A549 cells. (i) Analyses of NLRP3 expression (normalized to *β*-actin), *n* = 6, analyzed by two-way ANOVA with the Bonferroni post hoc test, ^∗∗∗∗^*P* < 0.0001 versus the normoxia group, ^###^*P* < 0.001 versus the hyperoxia group, ^&&&&^*P* < 0.0001 versus the hyperoxia + simvastatin (HS) group. (j, k) TNF-*α* and IL-6 levels in cells of different groups, *n* = 6, analyzed by two-way ANOVA with the Bonferroni post hoc test, ^∗∗∗∗^*P* < 0.0001 versus the normoxia group; ^###^*P* < 0.001 and ^####^*P* < 0.0001 versus the hyperoxia group; ^&&&&^*P* < 0.0001 versus the hyperoxia + simvastatin group.

A549 cells were treated with different simvastatin concentrations simultaneously with normoxia or hyperoxia for 48 h to choose the optimal therapeutic concentration. In Figure 7(a), simvastatin doses ranging from 2.5 to 15 *μ*M exerted lung-protective effects and the most obvious effect was observed at a dose of 5 *μ*M. As a result, 5 *μ*M simvastatin was employed in all subsequent cell experiments. Consistently with *in vivo* experiments (Figures 2–4), we found that high oxygen significantly reduced KLF2 expression and increased the expression of NLRP3 protein and inflammatory cytokines in A549 cells and simvastatin could reverse the effect of high oxygen. Interestingly, during the *in vitro* study, we added hyperoxia + simvastatin + KLF2 shRNA group (HS + KLF2shRNA) and found that KLF2 shRNA could significantly reduce these protective effects of simvastatin. As demonstrated in Figures 7(b)–7(d), hyperoxia reduced KLF2 levels in A549 cells, whereas simvastatin increased KLF2 expression in the hyperoxia group but not in KLF2-silenced group. In addition, hyperoxia increased NLRP3 levels in A549 cells, which was restored by simvastatin treatment but not in the KLF2-silenced group (Figures 7(h) and 7(i)). As indicated in Figures 7(j) and 7(k), simvastatin treatment can reduce hyperoxia-induced elevation of IL-6 and TNF-*α* but this therapeutic effect of simvastatin was abolished by KLF2 silencing.

## 4. Discussion

Bronchopulmonary dysplasia (BPD) is a common complication of premature birth [38]. Exposure to high-oxygen concentrations has been demonstrated to cause simplified lung development, leading to BPD [39]. Modelling BPD induced by high-oxygen levels is the most frequently used model, resulting in arrested lung growth, alveolar simplification, impaired blood vessel development, and abnormal pulmonary function. In this study, we used neonatal SD rats and A549 cells exposed to oxygen for 7 days and 48 hours, respectively, to establish a model of hyperoxic lung injury *in vivo* and *in vitro*.

Simvastatin is an HMG-CoA reductase inhibitor and is currently used as an anticholesterol drug. In addition, numerous recent studies have revealed that simvastatin has anti-inflammatory, antioxidant, and vascular protective effects [40, 41]. Tulbah et al. believed that anti-inflammatory, immunomodulatory, fibrinolytic, and antithrombotic activities and improvement of endothelial cell function of statins might make them a class of drugs for alternative treatments of chronic lung diseases [42]. Studies have indicated that simvastatin and atorvastatin can improve the health of patients with COPD and asthma by reducing pulmonary artery pressure and inflammatory mediators [43–46]. However, there is no report on whether simvastatin has anti-inflammatory or antioxidant effects on hyperoxia lung injury. Here, we investigated the potential protective mechanism of simvastatin in hyperoxia lung injury through *in vivo* and *in vitro* models and explored its possible mechanism. Simvastatin treatment could improve alveolar simplification caused by hyperoxia in neonatal rats while also decreasing oxidative stress and inflammation. In A549 cells exposed to high oxygen *in vitro*, simvastatin could promote cell survival and reduce inflammation. It is worth mentioning that we found that simvastatin higher than 20 *μ*M could inhibit the proliferation of A549 cells. This may be linked to the antiproliferative effect of simvastatin in lung diseases and its potential to treat lung cancer. Shang et al. found that simvastatin can inhibit the extracellular signal-regulated kinase (ERK) pathway, downregulate the expression of tumor necrosis factor *β* (TNF-*β*) receptor II, and inhibit the proliferation of A549 cells [47].

To further explore BPD pathogenesis and the possible mechanism of simvastatin, we examined the role of KLF2 in a hyperoxic lung injury model. Krüppel-like factor 2 (KLF2), alternatively referred to as lung KLF, is implicated in many biological processes, including inflammation [48]. KLF2 gene deletion can cause vascular maturation disorder and abnormal lung development in mice [22, 49]. KLF2 expression has been significantly reduced in many lung diseases, such as influenza virus and lipopolysaccharide-induced ALI, and KLF2 overexpression can significantly improve ALI [22]. Using *in vivo* and *in vitro* models, we investigated the protective effect of KLF2 on hyperoxic lung injury models and its potential mechanisms. Our results indicate that KLF2 expression was significantly decreased in lung tissues and A549 cells following hyperoxia exposure. Furthermore, we discovered that silencing KLF2 gene expression using KLF2 shRNA plasmid not only reduced cell activity under normal oxygen conditions but also aggravated hyperoxia-caused cell damage. In addition, KLF2 shRNA could increase NLRP3 and inflammatory cytokine expression. Therefore, our results suggest that KLF2 plays an important protective role in hyperoxia-induced lung injury.

Multiple evidences indicate that simvastatin is a strong inducer of KLF2 [30, 50, 51]. Statins can directly induce KLF2 expression by binding to the MEF2 transcription factor in the promoter region of KLF2 [52, 53]. To further confirm whether the protective effect of simvastatin against hyperoxia-induced lung injury is through the KLF2 pathway, we included the HS + KLF2 shRNA group in the *in vitro* experiment. Our study stated that after silencing the KLF2 gene during simvastatin treatment, the anti-inflammatory impact of simvastatin was reversed. The disadvantage is that we have only proved during *in vitro* experiments that simvastatin exerts its anti-inflammatory effect through the KLF2 pathway and we have not investigated whether the antioxidant effect of simvastatin is also correlated with KLF2. Simvastatin has been demonstrated to improve endothelial function by its antioxidant effect in many studies, possibly linked to inhibiting the mevalonic acid (MVA) pathway and isoprenoid synthases and deactivating nicotinamide adenine dinucleotide phosphate (NADPH) [54–56]. *In vivo* and *in vitro* experimental studies have demonstrated that simvastatin could reduce oxidative stress in many lung diseases by inhibiting the GTPases (Rac) pathway and inactivating the NADPH oxidase system at a cellular level, reducing ROS generation [54].

Although the results of this study indicate that simvastatin protects neonatal rats with hyperoxic lung injury, its clinical application and BPD should be further discussed. For most people, statins are safe and well tolerated. However, 30% of statins have been associated with intolerance, including the most prevalent muscle and liver toxicities [57]. The strengthening of childhood hypercholesterolemia screening has increased the use of statins in children [58]. In 2010, Cochrane systematic review evaluated the safety of statins in children. Compared with the control group, simvastatin was not found to have significant liver toxicity [58]. However, its long-term safety at this age has not been effectively confirmed. In addition, because statins have not been used clinically in neonates and the use of simvastatin in BPD may inhibit cholesterol synthesis in premature infants [59, 60], so, the application of simvastatin in neonatal bronchopulmonary dysplasia warrants additional investigation.

Our study has some limitations. (i) Because we only examined the effect of silencing KLF2 on hyperoxic lung injury and the therapeutic impact of simvastatin *in vitro*, *in vivo* inhibition of KLF2 should be included in future studies. (ii) Although this study found that knocking down KLF2 at the cellular level decreased alveolar epithelial cell activity and elevated inflammatory cytokines, we did not investigate whether KLF2 overexpression was protective. (iii) We have only employed *in vitro* experiments to confirm that simvastatin exerts its anti-inflammatory effect through the KLF2 pathway, but we did not investigate whether the antioxidant effect of simvastatin is also linked to KLF2. (iv) We selected the A549 cell line for *in vitro* experiments. Although it is a human-derived cell, the A549 cell line is derived from a 58-year-old male lung cancer cell. It may be different from neonatal lung epithelial cells, and additional research is required to rule out the effect of simvastatin on lung cancer.

## 5. Conclusion

In summary, this study demonstrated that simvastatin could ameliorate lung injury following hyperoxia exposure by stimulating KLF2 expression and suppressing NLRP3 inflammasome formation. While future clinical applications of simvastatin require additional research, it may be a promising treatment for BPD.

## Data Availability

The datasets used and/or analyzed during the current study are available from the corresponding author upon reasonable request.

## References

[1] Bhattacharya S., Mereness J. A., Baran A. M. (2020). Lymphocyte-specific biomarkers associated with preterm birth and bronchopulmonary dysplasia. *Frontiers in Immunology*.

[2] Collaco J. M., Romer L. H., Stuart B. D. (2012). Frontiers in pulmonary hypertension in infants and children with bronchopulmonary dysplasia. *Pediatric Pulmonology*.

[3] Abman S. H., Hansmann G., Archer S. L. (2015). Pediatric pulmonary hypertension: guidelines from the American Heart Association and American Thoracic Society. *Circulation*.

[4] de Wijs-Meijler D. P., Duncker D. J., Tibboel D. (2017). Oxidative injury of the pulmonary circulation in the perinatal period: Short- and long-term consequences for the human cardiopulmonary system. *Pulmonary Circulation*.

[5] Wang J., Dong W. (2018). Oxidative stress and bronchopulmonary dysplasia. *Gene*.

[6] Hummler J. K., Dapaah-siakwan F., Vaidya R. (2017). Inhibition of Rac1 signaling downregulates inflammasome activation and attenuates lung injury in neonatal rats exposed to hyperoxia. *Neonatology*.

[7] Liao J., Kapadia V. S., Brown L. S. (2015). The NLRP3 inflammasome is critically involved in the development of bronchopulmonary dysplasia. *Nature Communications*.

[8] Savani R. C. (2018). Modulators of inflammation in bronchopulmonary dysplasia. *Seminars in Perinatology*.

[9] Vaidya R., Zambrano R., Hummler J. K. (2017). Recombinant CCN1 prevents hyperoxia-induced lung injury in neonatal rats. *Pediatric Research*.

[10] Jiang L., Fei D., Gong R. (2016). CORM-2 inhibits TXNIP/NLRP3 inflammasome pathway in LPS-induced acute lung injury. *Inflammation Research*.

[11] Zhao W., Ma L., Cai C., Gong X. (2019). Caffeine inhibits NLRP3 inflammasome activation by suppressing MAPK/NF-*κ*B and A2aR signaling in LPS-induced THP-1 macrophages. *International Journal of Biological Sciences*.

[12] Tian Q., Xu M., He B. (2021). Histidine ameliorates elastase- and lipopolysaccharide-induced lung inflammation by inhibiting the activation of the NLRP3 inflammasome. *Acta Biochimica et Biophysica Sinica*.

[13] Ji J., Hou J., Xia Y., Xiang Z., Han X. (2021). NLRP3 inflammasome activation in alveolar epithelial cells promotes myofibroblast differentiation of lung-resident mesenchymal stem cells during pulmonary fibrogenesis. *Biochimica et Biophysica Acta (BBA) - Molecular Basis of Disease*.

[14] Jiang R., Xu J., Zhang Y., Zhu X., Liu J., Tan Y. (2021). Ligustrazine alleviate acute lung injury through suppressing pyroptosis and apoptosis of alveolar macrophages. *Frontiers in Pharmacology*.

[15] Zhang Q., Ran X., He Y., Ai Q., Shi Y. (2020). Acetate downregulates the activation of NLRP3 inflammasomes and attenuates lung injury in neonatal mice with bronchopulmonary dysplasia. *Frontiers in Pediatrics*.

[16] Chen S., Wu Q., Zhong D., Li C., Du L. (2020). Caffeine prevents hyperoxia-induced lung injury in neonatal mice through NLRP3 inflammasome and NF-*κ*B pathway. *Respiratory Research*.

[17] Pearson R., Fleetwood J., Eaton S., Crossley M., Bao S. (2008). Krüppel-like transcription factors: a functional family. *The International Journal of Biochemistry & Cell Biology*.

[18] Kuebler W. M. (2017). The flow-dependent transcription factor KLF2 protects lung vascular barrier function in acute respiratory distress syndrome. *American Journal of Respiratory and Critical Care Medicine*.

[19] Wani M. A., Wert S. E., Lingrel J. B. (1999). Lung Kruppel-like factor, a zinc finger transcription factor, is essential for normal lung development. *Journal of Biological Chemistry*.

[20] Turpaev K. T. (2020). Transcription Factor KLF2 and Its Role in the Regulation of Inflammatory Processes. *Biochemistry*.

[21] Sweet D. R., Fan L., Hsieh P. N., Jain M. K. (2018). Krüppel-like factors in vascular inflammation: mechanistic insights and therapeutic potential. *Frontiers in Cardiovascular Medicine*.

[22] Huang R. T., Meliton A., Oh M. J. (2017). Experimental lung injury reduces Krüppel-like factor 2 to increase endothelial permeability via regulation of RAPGEF3-Rac 1 signaling. *American Journal of Respiratory and Critical Care Medicine*.

[23] Saavedra M. T., Patterson A. D., West J. (2008). Abrogation of anti-inflammatory transcription factor LKLF in neutrophil-dominated airways. *American Journal of Respiratory Cell and Molecular Biology*.

[24] Shi J., Zhou X.-s. W., Du J.-f. (2018). KLF2 attenuates bleomycin-induced pulmonary fibrosis and inflammation with regulation of AP-1. *Biochemical and Biophysical Research Communications*.

[25] Sindi H. A., Russomanno G., Satta S. (2020). Author Correction: Therapeutic potential of KLF2-induced exosomal microRNAs in pulmonary hypertension. *Nature Communications*.

[26] Climent E., Benaiges D., Pedro-Botet J. (2021). Hydrophilic or Lipophilic Statins?. *Frontiers in Cardiovascular Medicine*.

[27] Margaritis M., Channon K. M., Antoniades C. (2014). Statins as regulators of redox state in the vascular endothelium: beyond lipid lowering. *Antioxidants & Redox Signaling*.

[28] Mihos C. G., Pineda A. M., Santana O. (2014). Cardiovascular effects of statins, beyond lipid-lowering properties. *Pharmacological Research*.

[29] Vogiatzi G., Oikonomou E., Siasos G. (2017). Statins and inflammation in cardiovascular disease. *Current Pharmaceutical Design*.

[30] Li H., Wang Y., Liu J. (2021). Endothelial Klf2-Foxp1-TGF*β* signal mediates the inhibitory effects of simvastatin on maladaptive cardiac remodeling. *Theranostics*.

[31] Zhang Y., Yin Y., Zhang W. (2021). Reactive oxygen species scavenging and inflammation mitigation enabled by biomimetic prussian blue analogues boycott atherosclerosis. *Journal of Nanobiotechnology*.

[32] Liu Z., Lai C.-h., Zhang X. (2019). Simvastatin ameliorates total liver ischemia/reperfusion injury via KLF2-mediated mechanism in rats. *Clinics and Research in Hepatology and Gastroenterology*.

[33] Sun X., Mathew B., Sammani S., Jacobson J. R., Garcia J. G. N. (2017). Simvastatin-induced sphingosine 1-phosphate receptor 1 expression is KLF2-dependent in human lung endothelial cells. *Pulmonary Circulation*.

[34] Li H. X., Liang X.-y., Wu J.-h., Yuan Y.-p., Gao Y., Cai S.-h. (2021). Simvastatin attenuates acute lung injury by activation of A2B adenosine receptor. *Toxicology and Applied Pharmacology*.

[35] Tu R. F., He Z. H., Tan X. W. (2021). Effects of simvastatin on pulmonary fibrosis and endothelial - mesenchymal transition in the pulmonary fibrosis tissue of rats.

[36] Tuuminen R., Nykänen A. I., Saharinen P. (2013). Donor simvastatin treatment prevents ischemia-reperfusion and acute kidney injury by preserving microvascular barrier function. *American Journal of Transplantation*.

[37] Konishi Y., Stegmüller J., Matsuda T., Bonni S., Bonni A. (2004). Cdh1-APC controls axonal growth and patterning in the mammalian brain. *Science*.

[38] Dumpa V., Bhandari V. (2021). Non-invasive ventilatory strategies to decrease bronchopulmonary dysplasia-where are we in 2021?. *Children*.

[39] Xuefei Y., Xinyi Z., Qing C. (2021). Effects of hyperoxia on mitochondrial homeostasis: are mitochondria the hub for bronchopulmonary dysplasia?. *Frontiers in Cell and Developmental Biology*.

[40] Moshiri M., Mehmannavaz F., Hashemi M., Yazdian-Robati R., Shabazi N., Etemad L. (2021). Evaluation of the efficiency of simvastatin loaded PLGA nanoparticles against acute paraquat-intoxicated rats. *European Journal of Pharmaceutical Sciences*.

[41] Rezaie-Majd A., Maca T., Bucek R. A. (2002). Simvastatin reduces expression of cytokines interleukin-6, interleukin-8, and monocyte chemoattractant protein-1 in circulating monocytes from hypercholesterolemic patients. *Arteriosclerosis, Thrombosis, and Vascular Biology*.

[42] Tulbah A. S., Ong H. X., Colombo P., Young P. M., Traini D. (2016). Could simvastatin be considered as a potential therapy for chronic lung diseases? A debate on the pros and cons. *Expert opinion on drug delivery*.

[43] Blamoun A. I., Batty G. N., DeBari V. A., Rashid A. O., Sheikh M., Khan M. A. (2008). Statins may reduce episodes of exacerbation and the requirement for intubation in patients with COPD: evidence from a retrospective cohort study. *International Journal of Clinical Practice*.

[44] Cowan D. C., Cowan J. O., Palmay R., Williamson A., Taylor D. R. (2010). Simvastatin in the treatment of asthma: lack of steroid-sparing effect. *Thorax*.

[45] Hothersall E. J., Chaudhuri R., McSharry C. (2008). Effects of atorvastatin added to inhaled corticosteroids on lung function and sputum cell counts in atopic asthma. *Thorax*.

[46] Lee T. M., Chen C.-C., Shen H.-N., Chang N.-C. (2009). Effects of pravastatin on functional capacity in patients with chronic obstructive pulmonary disease and pulmonary hypertension. *Clinical Science*.

[47] Shang L., Jia S.-S., Jiang H.-M., Wang H., Xu W.-H., Lv C.-J. (2015). Simvastatin downregulates expression of TGF-*β*RII and inhibits proliferation of A549 cells via ERK. *Tumour Biology*.

[48] Chiplunkar A. R., Curtis B. C., Eades G. L. (2013). The Krüppel-like factor 2 and Krüppel-like factor 4 genes interact to maintain endothelial integrity in mouse embryonic vasculogenesis. *BMC Developmental Biology*.

[49] Jha P., Das H. (2017). KLF2 in regulation of NF-*κ*B-mediated immune cell function and inflammation. *International Journal of Molecular Sciences*.

[50] Liu Z., Zhang X., Xiao Q. (2017). Pretreatment donors after circulatory death with simvastatin alleviates liver ischemia reperfusion injury through a KLF2-dependent mechanism in rat. *Oxidative Medicine and Cellular Longevity*.

[51] Parmar K. M., Nambudiri V., Dai G., Larman H. B., Gimbrone M. A., García-Cardeña G. (2005). Statins exert endothelial atheroprotective effects via the KLF2 transcription factor. *Journal of Biological Chemistry*.

[52] Marschall J. S., Wilhelm T., Schuh W., Huber M. (2012). MEK/Erk-based negative feedback mechanism involved in control of Steel Factor-triggered production of Krüppel-like factor 2 in mast cells. *Cellular Signalling*.

[53] Sen-Banerjee S., Mir S., Lin Z. (2005). Kruppel-like factor 2 as a novel mediator of statin effects in endothelial cells. *Circulation*.

[54] Chen W., Pendyala S., Natarajan V., Garcia J. G. N., Jacobson J. R. (2008). Endothelial cell barrier protection by simvastatin: GTPase regulation and NADPH oxidase inhibition. *American Journal of Physiology. Lung Cellular and Molecular Physiology*.

[55] Sun X. F., Wang L. L., Wang J. K. (2007). Effects of simvastatin on lung injury induced by ischaemia-reperfusion of the hind limbs in rats. *The Journal of International Medical Research*.

[56] Xavier A. M., Serafim K. G. G., Higashi D. T. (2012). Simvastatin improves morphological and functional recovery of sciatic nerve injury in Wistar rats. *Injury*.

[57] Attalah Nee Rezkallah C., Thongkum A., Zhu C., Chen Q. M. (2020). Resveratrol for protection against statin toxicity in C2C12 and H9c2 cells. *Journal of Biochemical and Molecular Toxicology*.

[58] Vuorio A., Kuoppala J., Kovanen P. T. (2019). Statins for children with familial hypercholesterolemia. *The Cochrane Database of Systematic Reviews*.

[59] Dias I. H. K., Milic I., Lip G. Y. H., Devitt A., Polidori M. C., Griffiths H. R. (2018). Simvastatin reduces circulating oxysterol levels in men with hypercholesterolaemia. *Redox Biology*.

[60] Rossebø A. B., Pedersen T. R., Boman K. (2008). Intensive lipid lowering with simvastatin and ezetimibe in aortic stenosis. *The New England Journal of Medicine*.

